# Environmental enrichment attenuates chronic stress–induced disruptions in the gut microbiota, intestinal barrier, and brain

**DOI:** 10.1371/journal.pone.0355857

**Published:** 2026-08-14

**Authors:** Seung Ju Kang, Hyeong Ju Nam, Min Kyung Song

**Affiliations:** College of Nursing Science, Kyung Hee University, Seoul, Republic of Korea; Public Library of Science, UNITED KINGDOM OF GREAT BRITAIN AND NORTHERN IRELAND

## Abstract

Major depressive disorder is a prevalent mood disorder characterized by affective and cognitive impairment. Alterations in gut microbiota have been implicated in its pathophysiology, as stress-related microbial changes can disrupt intestinal barrier integrity and promote inflammatory activation along the microbiota–gut–brain axis. Although environmental enrichment (EE) has been shown to exert beneficial effects on stress-related behaviors, its influence on gut microbiota under chronic stress conditions remains unclear. Here, we examined the effects of EE on depressive-like behavior, stress-related molecular markers in the brain, gut microbiota composition, and intestinal barrier function in a chronic unpredictable mild stress (CUMS) mouse model. Following CUMS exposure for 5 weeks, mice were housed in either a standard environment or EE for 4 weeks. EE significantly reduced immobility time in the tail suspension test and restored tryptophan hydroxylase‑positive cell numbers in the dorsal raphe nucleus while reversing glucocorticoid receptor‑positive cell expression in the hippocampus. Microbiota analysis revealed that CUMS elevated alpha diversity and altered beta diversity, accompanied by increases in stress‑associated taxa—including *Firmicutes*, *Desulfobacterota*, *Patescibacteria*, and *Ruminococcus*—and reductions in beneficial taxa such as *Akkermansia*. EE prevented the CUMS-induced alterations, reestablishing microbial diversity and community structure toward control levels and partially reversing the taxonomic changes. In the colon, CUMS reduced claudin‑1 expression and increased IL‑6 and NF‑κB protein levels, reflecting impaired barrier integrity and enhanced inflammatory responses. EE attenuated the CUMS-induced intestinal disruptions, restoring tight junction protein expression and reducing inflammatory markers. In conclusion, EE may serve as a non‑pharmacological intervention capable of stabilizing the microbiota–gut–brain axis in depressive‑like states.

## Introduction

Major depressive disorder (MDD) is a prevalent psychiatric condition characterized by persistent low mood, anhedonia, cognitive impairment, and disturbances in sleep and appetite [1]. Its etiology is multifactorial, arising from complex interactions among biological, genetic, psychological, and environmental determinants [2]. Chronic stress is recognized as a major contributor to MDD pathophysiology by dysregulating the hypothalamic–pituitary–adrenal (HPA) axis and disrupting neuroendocrine and immune homeostasis [3–6]. Growing evidence further demonstrates that chronic stress–induced alterations extend beyond the central nervous system, affecting the microbiota–gut–brain axis, which may offer novel targets for therapeutic intervention in depression [7].

Chronic stress disrupts gut microbiota homeostasis and induces dysbiosis [8,9]. This imbalance is characterized by alterations in microbial diversity and community composition and deterioration of microbial metabolic capacity, notably a decline in butyrate-producing bacteria. Butyrate, a key histone deacetylase inhibitor, plays a pivotal role in maintaining intestinal barrier function [10]. Its reduction leads to histone deacetylation and transcriptional repression of tight junction proteins such as Zonula Occludens‑1 (ZO‑1) and Occludin [11]. Decreased expression of these proteins compromises epithelial barrier integrity, leading to increased intestinal permeability, or “leaky gut” [12]. Under these conditions, lipopolysaccharides and other bacterial products translocate into systemic circulation [13]. This cascade involves increased secretion of pro‑inflammatory cytokines, including interleukin‑6 (IL‑6) and interleukin‑1β (IL‑1β), as well as activation of key inflammatory signaling mediators, such as inducible nitric oxide synthase (iNOS) and nuclear factor‑κB (NF‑κB) [14,15].

Peripheral inflammation directly influences central neurotransmission. Elevated circulating cytokines upregulate indoleamine 2,3‑dioxygenase, diverting tryptophan toward the kynurenine pathway and reducing its availability for serotonin synthesis [16]. This process not only decreases serotonergic neurotransmission but also produces neuroactive metabolites that may exacerbate depressive symptoms [17]. These processes underscore the involvement of the microbiota–gut–brain axis in chronic stress–related alterations relevant to MDD, highlighting potential targets for anti-inflammatory and neuroprotective therapies.

Among the available animal models of stress-induced depression, the Chronic Unpredictable Mild Stress (CUMS) paradigm is one of the most widely used [18]. CUMS subjects rodents to a randomized sequence of mild and unpredictable stressors—such as food or water deprivation, cage tilting, or altered light–dark cycles—administered over several weeks to prevent habituation [19]. This paradigm reproduces key characteristics of human stress-related depression, including HPA axis hyperactivation, impaired neuroplasticity, reduced Tryptophan hydroxylase (TPH) expression, and disturbances in gut microbial composition [20–22]. Consequently, CUMS provides a robust experimental framework for investigating mechanisms underlying MDD and for evaluating interventions that influence both central and peripheral components of the disorder.

Environmental enrichment (EE) is a non-pharmacological intervention that exerts broad effects on both central neural processes and peripheral physiology [23]. EE involves enhanced housing conditions that provide increased sensory, cognitive, motor, and social stimulation [24]. In animal models, EE has been demonstrated to promote neurogenesis, facilitate synaptic plasticity, and reduce HPA axis hyperactivity [25,26]. Evidence further indicates that EE influences the microbiota–gut–brain axis. Reported effects include increased microbial diversity, restoration of beneficial taxa such as Lactobacillus and Bifidobacterium, augmented short-chain fatty acid production, and improved intestinal barrier integrity, which collectively attenuate stress-induced inflammatory responses [27,28]. These findings suggest that EE modulates interconnected biological pathways disrupted by chronic stress and may serve as a practical approach for addressing both central and peripheral components of stress-related pathology.

Accordingly, the present study examined whether EE mitigates the effects of chronic stress–related disruptions on the microbiota–gut–brain axis in a CUMS mouse model. We examined how EE influences stress-induced alterations in depressive-like behaviors, gut microbial composition, and neurobiological conditions. Our study aimed to evaluate its potential as a non-pharmacological strategy for alleviating depression-related changes.

## Materials and methods

### Animals

In total, 23 male C57BL/6J mice (7 weeks, 22−26 g) were obtained from Young Bio (Seongnam, Korea). Animals were acclimated for one week prior to experimentation and housed under controlled conditions (25 ± 2 °C, 50 ± 10% humidity, 12-h light/dark cycle) with *ad libitum* access to food and water. Sample sizes were determined using G*Power software (version 3.1). With parameters for an F-test (one-way ANOVA) set to an effect size of 0.75, an alpha level of 0.05, and a power of 0.8 across 3 groups, the analysis indicated a required minimum total sample size of 21 mice. To satisfy this statistical requirement while maximizing the social interaction component, which is crucial to the EE paradigm, we allocated a larger cohort to the EE group. Consequently, the final number of mice used in this study was 23. All procedures adhered to the NIH Guide for the Care and Use of Laboratory Animals and relevant institutional regulations, and were approved by the Kyung Hee University Institutional Animal Care and Use Committee (IACUC protocol no. KHSASP-24–017).

### Experimental designs

**Fig 1:**
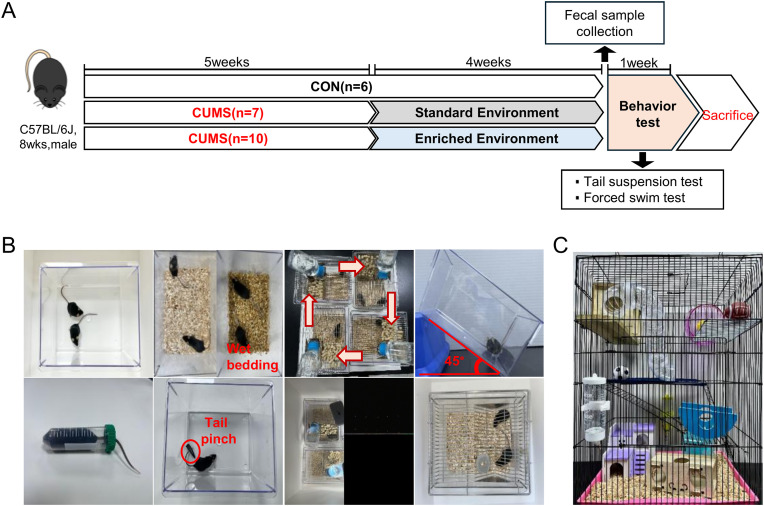
(A) Experimental design. (B) Stressor used in the Chronic Unpredictable Mild Stress (CUMS) model. (C) Environmental enrichment cage.

Following acclimation, mice were randomly assigned to one of three groups: (1) Control group (CON, n = 6); (2) CUMS-exposed group housed in a Standard Environment (CUMS+SE, n = 7); (3) a CUMS-exposed group housed in an Enriched Environment (CUMS+EE, n = 10). To ensure unbiased allocation, the randomization was performed by an independent researcher who was not involved in the behavioral experiments or data collection. Stress-exposed groups underwent a 5-week CUMS protocol to induce depressive-like behaviors, whereas the CON group remained undisturbed. After completion of the CUMS regimen, mice in the SE and EE groups were transferred to their respective housing condition for a 4-week environmental intervention. Fecal samples were collected at the end of the intervention for microbiome analysis, followed by behavioral testing to assess depression-like phenotypes. On the day after the final behavioral test, mice were deeply anesthetized with 5% isoflurane in an induction chamber. To ensure an adequate depth of anesthesia, the absence of a withdrawal reflex was confirmed by a strong tail pinch. Subsequently, cervical dislocation was performed to ensure a rapid and painless sacrifice. Immediately after euthanasia, brain and gut tissues were harvested and processed for downstream analyses (Fig 1A). Throughout the experimental period, every effort was made to alleviate potential suffering, including regular health monitoring and the provision of a stable, stress-free environment. For all subsequent molecular analyses, the experimenters remained blinded to the group assignments to prevent bias during data collection and quantification.

### Chronic unpredictable mild stress (CUMS)

Depression-like behaviors were induced by using a validated CUMS protocol consisting of eight mild stressors adapted from previous studies [29–31]. Two different stressors were presented daily in a randomized order for 5 weeks, with no stressor repeated on consecutive days. The stressors included (Fig 1B):

- **Empty Cage:** 4 h housing without bedding.
- **Wet Bedding:** 4 h exposure to bedding moistened with ~450 mL of water.
- **Soiled Bedding Exposure:** 3 h housing in bedding previously used by another cage.
- **45° Cage Tilt:** 4 h tilt of the home cage at a 45° angle.
- **Restraint Stress:** 1 h placement in a ventilated restrainer tube (4 cm × 12 cm).
- **Tail Pinch:** Mild tail pinch for 5 min using a clip applied to the tail tip.
- **Light/Dark Cycle Reversal:** 24 h inversion of the standard light cycle.
- **Food/Water Deprivation:** 24 h removal of food and water.

### Environmental intervention

Environmental conditions were manipulated for 4 weeks, following the CUMS protocol, to evaluate the modulatory effects of a differential housing environment [23,24,32]. The CON and CUMS + SE groups remained in standard cages (23 × 17 × 13 cm) and were housed at 3–4 mice per cage throughout the study. In contrast, the CUMS + EE group was transferred to a larger enriched cage (42 × 30 × 60 cm), where 10 mice were co-housed per cage. Although the absolute number of mice per cage was higher in the EE group, the significantly larger dimensions and multi-level design of the enriched cages provided greater overall space per animal (lower spatial density) compared to the standard cages. This specific co-housing setup was designed to enhance physical activity and social interaction. Enriched cages featured multi-level wire-mesh structures with vertical climbing surfaces connected by ladders and bars, along with various enrichment items such as tunnels, shelters, stairs, and running wheels (Fig 1C). To minimize habituation, enrichment items were rearranged weekly, and food and water access points were changed daily to promote exploratory and foraging behaviors.

### Tail suspension test

The tail suspension test (TST) was conducted to assess behavioral despair, following established protocol [33]. Mice were suspended by securing approximately 1 cm of the tail tip to a horizontal bar positioned 30 cm above the surface. Each trial lasted 6 min and was recorded using a video camera. Immobility—defined as the absence of voluntary limb and head movements except those required for minimal postural maintenance—was quantified. Two investigators, blinded to group assignments, independently scored immobility time, and their scores were averaged.

### Forced swim test

The forced swim test (FST) was performed to evaluate depressive-like behavior, following standard methodology [34]. Mice were individually placed into a transparent cylindrical tank (20 cm diameter, 100 cm height) filled with water (50 cm depth) maintained at 25 ± 2 °C, preventing animals from supporting themselves on the tank bottom. Each mouse was tested for 8 min, and behavior was video-recorded. Immobility was defined as the absence of active swimming or struggling, excluding minimal movements necessary to keep the head above water. Immobility time was scored by two independent investigators blinded to group allocation, and mean values were used for analysis.

### Immunohistochemistry

Whole brain tissues were post-fixed in 4% paraformaldehyde (PFA) overnight at 4°C, cryoprotected in 30% sucrose, and coronally sectioned at a thickness of 30 µm using a freezing microtome (CM3050 S Cryostat; Leica, Wetzlar, Germany). The hippocampal CA1 region and dorsal raphe nucleus were selected for analysis, and free-floating immunohistochemistry was performed. Sections were first incubated in 0.3% hydrogen peroxide in 1X PBS for 15 min at room temperature (RT) to quench endogenous peroxidase activity, followed by blocking in 1% bovine serum albumin (BSA) in 1X PBS for 1 h at RT. Tissues were then incubated overnight at 4°C with primary antibodies against TPH (1:1000, AB1541; MilliporeSigma, Hessen, Germany) and glucocorticoid receptor (GR, 1:500, #3660; Cell signaling, MA, USA) diluted in 1X PBS containing 0.3% Triton X-100 and 0.5% BSA. After washing, sections were incubated with biotinylated anti-sheep or anti-rabbit secondary antibodies (1:1000, Vector Laboratories, CA, USA) for 1 h at RT, followed by incubation with the avidin–biotin complex (ABC Elite Kit; Vector Laboratories, CA, USA) for 1 h at RT. Visualization was achieved using 3,3′-diaminobenzidine (DAB; Vector Laboratories, CA, USA). Stained sections were mounted on slides, coverslipped, and imaged using a bright-field microscope (Olympus BX51, Tokyo, Japan). Cell counts were performed by two independent investigators blinded to group assignments, and mean values were used for statistical analysis.

### Western blot

Colon tissues were collected immediately after sacrifice and flash-frozen on dry ice. Samples were homogenized in ice-cold radioimmunoprecipitation assay (RIPA) buffer supplemented with protease and phosphatase inhibitors (BIOPREP-24R; Allsheng, Hangzhou, China). Lysates were centrifuged to remove debris, and equal amounts of protein (10 µg) were mixed with 4X Laemmli sample buffer and heat-denatured at 95°C for 5 min. Proteins were separated by 8–12% SDS-PAGE and transferred onto polyvinylidene difluoride (PVDF) membranes. Membranes were blocked with 5% skim milk in Tris-buffered saline containing 0.1% Tween-20 (TBS-T), followed by overnight incubation with primary antibodies against interleukin-6 (IL-6, 1:1000, #12912, Cell signaling, MA, USA), NF-κB (1:1000, #8242, Cell Signaling, MA, USA), tumor necrosis factor-α (TNF-α, 1:1000, #11948, Cell Signaling, MA, USA), claudin-1(1:1000, 37–4900, Invitrogen, CA, USA), and occludin (1:1000, #91131, Cell Signaling, MA, USA), and β-actin (1:20,000, sc-47778, Santa Cruz, CA, USA). After washing, horseradish peroxidase (HRP)-conjugated anti-mouse or anti-rabbit secondary antibodies (1:5000, Vector Laboratories, CA, USA) were applied for 1 h at RT. Immunoreactive bands were detected using an enhanced chemiluminescence system (ChemiDoc Imaging System; Bio-Rad, CA, USA) and quantified using Image Lab software (Bio-Rad, CA, USA).

### Reverse transcriptional polymerase chain reaction

Total RNA was extracted from colon tissues using TRIzol reagent (Invitrogen, CA, USA) and chloroform. cDNA synthesis was performed with 1 µg of RNA using the amfiRivert cDNA Synthesis Platinum Master Mix (GenDEPOT, TX, USA) containing oligo(dT) primers, according to the manufacturer’s instructions. All primer sequences used for PCR analyses are presented in Table 1. Target gene expression was assessed by conventional PCR using the amfiSure PCR One-Step Master Mix (GenDEPOT, TX, USA) in a final reaction volume of 16 µL. PCR products were separated on 1.5% agarose gels, stained with nucleic acid dye (StaySafe; Real Biotech Corporation, Taiwan), and visualized under UV illumination using the ChemiDoc Imaging System (Bio-Rad, CA, USA). Band intensities were quantified with Image Lab software (Bio-Rad, CA, USA).

**Table 1:** List of primers used in RT-PCR.

Gene	Primer sequence (5’-3’)	
***CLDN1***	Forward	CCACCATTGGCATGAAGTGC
	Reverse	AGAGGTTGTTTTCCGGGGAC
***IL1B***	Forward	TGCCACCTTTTGACAGTGATG
	Reverse	AAGGTCCACGGGAAAGACAC
***NOS2***	Forward	CCTCCTCCACCCTACCAAGT
	Reverse	CACCCAAAGTGCTTCAGTCA
***NF-ΚB***	Forward	ACCATGGCAGACGATGATCC
	Reverse	TGCAAATTTTGACCTGTGGGT
***ACTB***	Forward	CGTTGACATCCGTAAAGACC
	Reverse	AACAGTCCGCCTAGAAGCAC

### 16S rRNA sequencing analysis

To assess the effects of chronic stress and environmental intervention on the gut microbiome, fecal samples were collected from each group at the end of the study. Samples were submitted to Oneomics (Gimpo, Korea) for genomic DNA extraction and sequencing. Sequencing was performed using the Oxford Nanopore Technologies (ONT) platform. To minimize potential batch effects, all samples were processed simultaneously using the same DNA extraction kits, PCR amplification protocols, and sequencing runs. The hypervariable V3–V4 regions of the bacterial 16S rRNA gene were amplified using universal primers 341F and 805R. Raw sequencing data were processed using the CLC metagenomics analysis pipeline. Chimera sequences were removed to eliminate artifactual reads, and the remaining high-quality sequences were clustered into operational taxonomic units (OTUs) at a 97% similarity threshold. Representative OTU sequences were taxonomically assigned by alignment against the SILVA 16S rRNA database (v138.1). The average sequencing depth was approximately 24,000 reads per sample. To correct for variations in sequencing depth and ensure equitable comparisons, the OTU abundance table was rarefied to an even depth of 15,000 reads per sample prior to downstream analysis. Alpha-diversity (within-sample diversity) and beta-diversity (between-sample dissimilarity) metrics were calculated to evaluate microbial community structure, followed by comparative analyses between experimental groups.

### Statistical analysis

Statistical analyses were performed using GraphPad Prism 10.3.1. Prior to parametric analyses, the normality of the data distribution was evaluated using the Shapiro-Wilk test, and the homogeneity of variance was assessed using the Brown-Forsythe test. Variables that met these assumptions were analyzed using a one-way analysis of variance (ANOVA) followed by Tukey’s post hoc test. Beta diversity was represented by a Principal Coordinate Analysis (PCoA) Emperor plot based on a Bray–Curtis distance matrix, and statistical differences in microbial community composition among groups were assessed using permutational multivariate analysis of variance (PERMANOVA). All data are presented as mean ± Standard Error of the Mean (SEM), and statistical significance was set at *p* < 0.05.

## Results

### EE alleviates the depressive-like behavior in the CUMS mouse model

**Fig 2:**
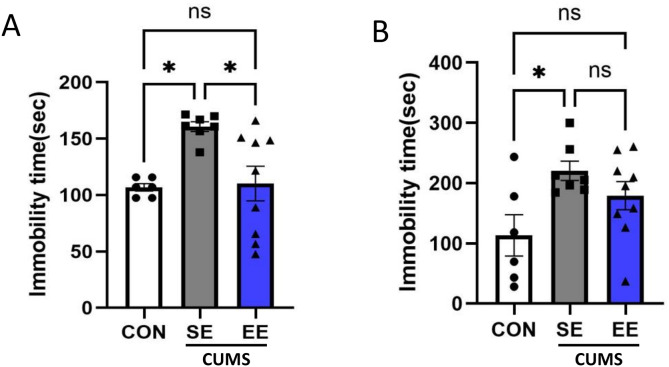
Effects of EE on depressive-like behaviors assessed by the tail suspension test (TST) and forced swim test (FST). Representative photographs of the results in two behavior tests: (A) TST and (B) FST. Data are presented as the mean ± S.E.M. (n = 6 for CON, n = 7 for CUMS+SE, and n = 9 for CUMS+EE). Statistical significance was determined by one-way ANOVA, followed by Tukey’s post-hoc test (* *p* < 0.05, ns: not significant).

The TST and FST were performed to evaluate depressive-like behaviors and to determine whether EE ameliorated CUMS-induced behavioral deficits. As shown in Fig 2, the CUMS+SE group exhibited significantly increased immobility times in both the TST and FST compared with the CON group, indicating successful induction of depressive-like behavior by the CUMS procedure (TST: F(2, 19) = 6.788, *p* = 0.006; FST: F(2, 19) = 4.156, *p* = 0.032). The CUMS+SE group showed substantially longer immobility times than the CON group in both the TST (*p* = 0.014) and FST (*p* = 0.025). Importantly, EE intervention significantly reduced TST immobility times compared with the CUMS+SE group (*p* = 0.011), with levels approaching those observed in the CON group. These findings suggest that EE may alleviate CUMS-induced depressive-like behavior in mice.

### EE restores TPH expression in the dorsal raphe and reduces CUMS-induced GR upregulation in the hippocampus

**Fig 3:**
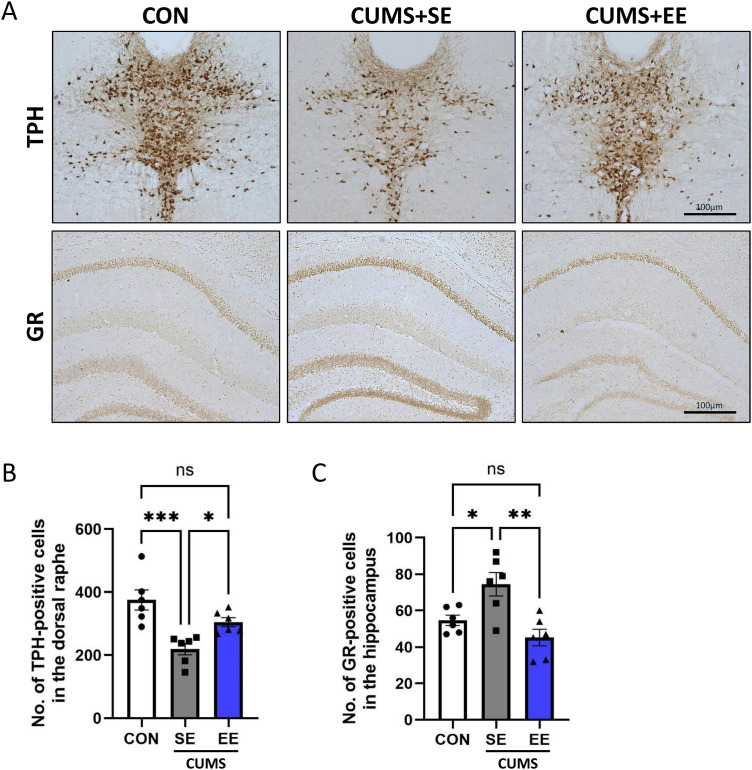
Effects of EE on TPH and GR expression in the dorsal raphe nucleus and hippocampus. (A) Representative micrographs of TPH and GR immunostaining in the dorsal raphe and hippocampus. (B) The number of TPH-positive cells in the dorsal raphe. (C) The number of GR-positive cells in the hippocampus. Data are presented as the mean ± S.E.M. (n = 6 per group). Statistical significance was determined by one-way ANOVA, followed by Tukey’s post-hoc test (* *p* < 0.05, ** *p* < 0.01, *** *p* < 0.001, ns: not significant). Scale bar: 100 μm.

Immunohistochemistry was performed to examine neurobiological alterations induced by CUMS and the modulatory effects of EE, with a focus on TPH expression in the dorsal raphe and GR expression in the hippocampus (Fig 3A). TPH, the rate-limiting enzyme in serotonin synthesis, is known to decrease under depressive-like conditions. A significant difference in TPH expression was observed among the groups (F(2, 15) = 11.840, *p* < 0.001) (Fig 3B). The CUMS+SE group showed a marked reduction in TPH-positive cells compared with the CON group (*p* < 0.001), indicating diminished serotonergic activity following CUMS exposure. In contrast, the CUMS+EE group exhibited a significant restoration of TPH-positive cell counts, reaching values comparable to those of the CON group (*p* = 0.104), suggesting that EE effectively counteracts the CUMS-induced reductions in serotonin synthesis.

GR expression, an indicator of HPA axis activation, also differed significantly among groups (F(2, 15) = 9.565, *p* = 0.002) (Fig 3C). GR-positive cells were substantially elevated in the CUMS+SE group relative to the CON group (*p* = 0.028), reflecting heightened glucocorticoid signaling induced by CUMS. Notably, GR expression was significantly reduced in the CUMS+EE group (*p* = 0.002), demonstrating that EE attenuates CUMS-associated neuroendocrine dysregulation.

### EE ameliorates CUMS‑induced changes in gut microbiota

**Fig 4:**
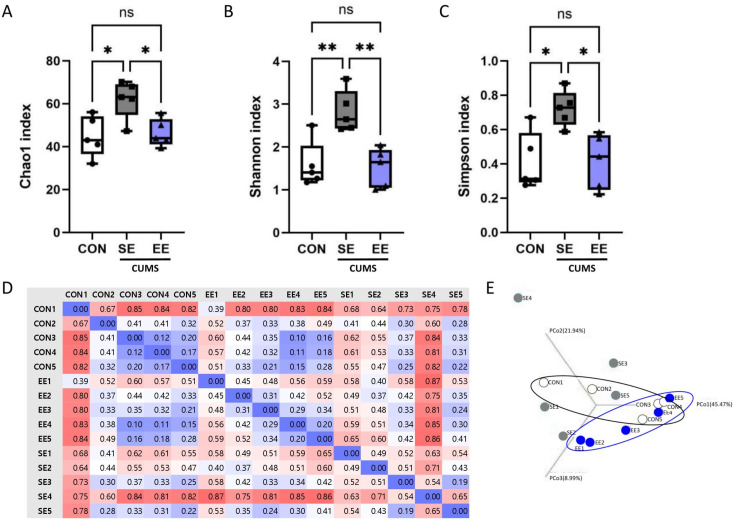
Effect of EE on gut microbiota diversity in CUMS mice. (A–C) Alpha diversity indices, including (A) Chao1 richness, (B) Shannon diversity, and (C) Simpson diversity. Data are presented as box-and-whisker plots indicating the median, interquartile range, and minimum–maximum values (n = 5 per group). (one-way ANOVA followed by Tukey’s test, * *p* < 0.05, ** *p* < 0.01, ns: not significant). (D-E) Beta diversity analysis. (D) Bray–Curtis distance heatmap illustrating within- and between-group dissimilarities. (E) PCoA plotted from Bray–Curtis distances showing group-level separation (n = 5 per group). Statistical differences in community composition were evaluated using PERMANOVA.

To determine whether EE effects are associated with alterations in gut microbiota composition, 16S rRNA sequencing was performed on fecal samples from each group. Alpha diversity analysis showed significant differences among the three groups(F = 6.476, *p* = 0.012 for Chao1; F = 11.020 for Shannon, *p* = 0.002; F = 7.350, *p* = 0.008 for Simpson). The Chao1 richness index was significantly increased in the CUMS+SE group compared with both the CON and CUMS+EE groups (*p* = 0.018 vs CON; *p* = 0.029 vs CUMS+EE), while CON and EE showed a similar pattern (Fig 4A). Likewise, the Shannon and Simpson diversity indices were markedly higher in the CUMS+SE group relative to both the CON and CUMS+EE groups (Shannon: *p* = 0.005 vs. CON, *p* = 0.004 vs. CUMS+EE; Simpson: *p* = 0.015 vs. CON, *p* = 0.016 vs. CUMS+EE), and CON and EE again exhibited comparable diversity levels (Figs 4B and C). Our data suggest that CUMS altered microbial richness and evenness compared with the control group, whereas EE intervention resulted in alpha-diversity profiles that were more comparable to those of the controls. Beta diversity analysis using the Bray–Curtis distance matrix further demonstrated distinct microbial community structures among the groups. The heatmap of pairwise Bray–Curtis distances showed group-specific clustering (Fig 4D). Principal Coordinate Analysis (PCoA) illustrated clear separation among groups, and PERMANOVA confirmed significant differences in microbial community composition (*p* = 0.014) (Fig 4E). Overall, these results show that CUMS significantly modifies gut microbiota composition. In contrast, EE attenuates these alterations and maintains a community structure similar to the control group.

**Fig 5:**
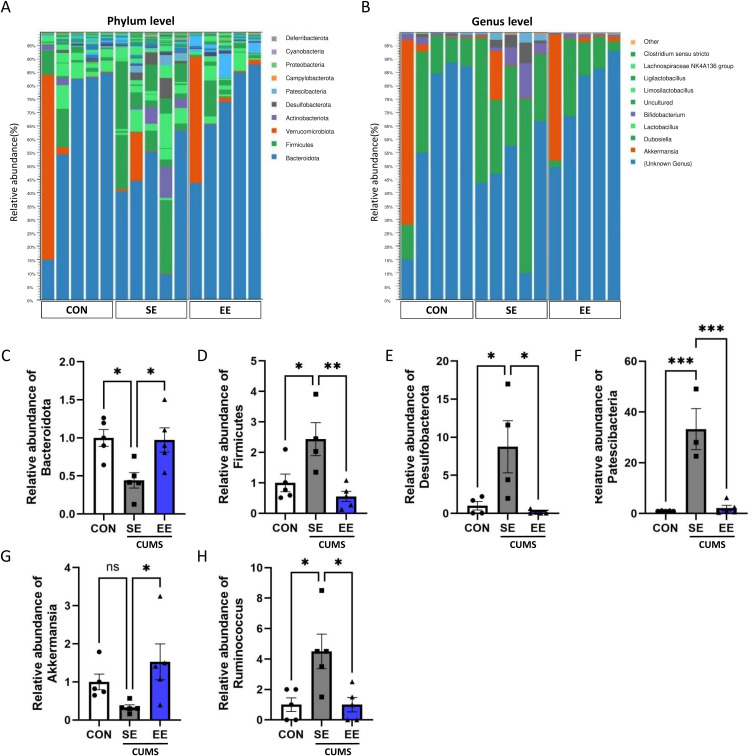
Effect of EE on gut microbiota composition in CUMS mice. (A) Relative abundance of major bacterial phyla. (B) Relative abundance of dominant genera. (C–H) Relative abundance of selected phyla and genera, including *Bacteroidota* (C), *Firmicutes* (D), *Desulfobacterota* (E), *Patescibacteria* (F) at the phylum level, *Akkermansia* (G), and *Ruminococcus* (H) at the genus level. Data are presented as the mean ± S.E.M. (n = 5 per group). Statistical significance was determined by one-way ANOVA (with F-statistics ranging from 3.99 to 27.90 for significant taxa), followed by Tukey’s post-hoc test (* *p* < 0.05, ** *p* < 0.01, *** *p* < 0.001, ns: not significant).

Taxonomic profiling revealed substantial alterations in gut microbial composition among the three groups. At the phylum level, the CUMS+SE group showed a decreased relative abundance of *Bacteroidota* accompanied by increased abundances of *Firmicutes*, *Desulfobacterota*, and *Patescibacteria*. These CUMS-induced alterations were attenuated in the CUMS+EE group, whose microbial composition more closely resembled that of the CON group (Fig 5A). A similar pattern was observed at the genus level, where SE mice displayed marked differences, while EE mice remained closer to the control profile (Fig 5B). Quantitative analyses confirmed these compositional changes. The relative abundance of *Bacteroidota* was significantly reduced in the CUMS+SE group compared with CON and EE (*p* = 0.023 vs. CON, *p* = 0.030 vs. CUMS+EE), while EE maintained levels similar to CON (Fig 5C). *Firmicutes*, *Desulfobacterota*, and *Patescibacteria* abundance were significantly elevated in the CUMS+SE group compared with both the CON and CUMS+EE groups (*Firmicutes*: *p* = 0.032 vs. CON, *p* = 0.007 vs. CUMS+EE; *Desulfobacterota*: *p* = 0.040 vs. CON, *p* = 0.018 vs. CUMS+EE; *Patescibacteria*: *p* < 0.001 vs. CON, *p* < 0.001 vs. CUMS+EE) (Figs 5D-F). *Akkermansia* abundance was reduced in the CUMS+SE group but was restored following EE intervention (*p* = 0.038 vs. CUMS+EE) (Fig 5G). Conversely, *Ruminococcus* levels were significantly elevated in the CUMS+SE group (*p* = 0.017 vs. CON) and were reduced to control-like levels in the CUMS+EE group (*p* = 0.017 vs. CUMS+SE) (Fig 5H). Overall, EE intervention shows the potential to mitigate CUMS-induced gut microbiota dysbiosis by supporting the partial restoration of the microbial community.

### EE increases tight junction proteins expression in the CUMS mouse model

**Fig 6:**
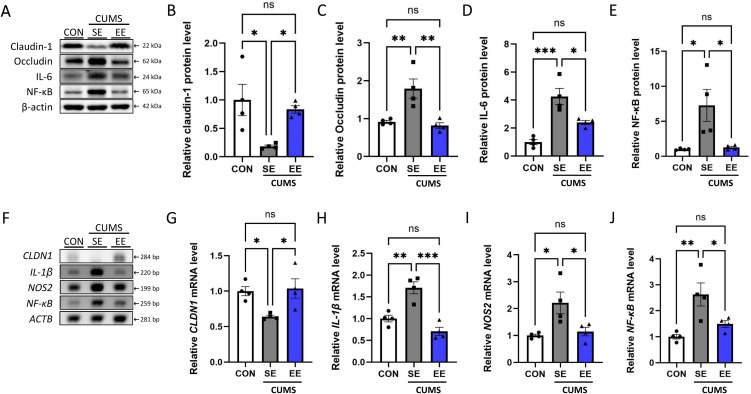
Effect of EE on tight junction proteins and inflammatory markers in CUMS mice. (A) Representative Western blot images of claudin‑1, occludin, IL‑6, and NF‑κB in colonic tissue. (B–E) Quantification of protein expression levels. (F) Representative RT‑PCR bands for *CLDN1*, *IL‑1β*, *NOS2*, and *NF‑κB*. (G–J) Relative mRNA expression levels. Data are presented as the mean ± S.E.M. (n = 4 per group). Statistical significance was determined by one-way ANOVA followed by Tukey’s post-hoc test (* *p* < 0.05, ** *p* < 0.01, *** *p* < 0.001, ns: not significant).

To determine whether CUMS disrupts intestinal barrier integrity, the expression of key tight junction proteins was assessed in gut tissues (Fig 6). Western blot analysis revealed a significant difference in claudin-1 levels among the groups (F(2, 9) = 7.021, *p* = 0.010). Claudin-1 expression was markedly reduced in the CUMS+SE group compared with the CON group (*p* = 0.009). EE intervention restored claudin-1 expression to control levels (*p* = 0.427) (Fig 6B), indicating its protective role. Regarding occludin levels, another key protein, there was a significant difference across the groups (F(2, 9) = 13.480, *p* = 0.002). Specifically, occludin expression was significantly increased in the CUMS+SE group compared to the CON group (*p* = 0.006) and EE treatment significantly attenuated this elevation (*p* = 0.003), bringing occludin expression closer to control levels (*p* = 0.857) (Fig 6C). RT-PCR revealed a significant group effect on *CLDN1* mRNA levels (F(2, 9) = 6.227, *p* = 0.020) (Fig 6F). Notably, the CUMS+SE group exhibited a marked decrease in *CLDN1* expression relative to the CON group (*p* = 0.043), which was subsequently restored in the EE-treated group (*p* = 0.027) (Figs 6F and G).

### EE decreases intestinal inflammatory responses in the CUMS mouse model

Western blot analysis and RT-PCR were performed to examine intestinal inflammatory responses associated with CUMS-induced depressive-like behavior to evaluate the anti-inflammatory effects of EE. The expression levels of pro-inflammatory cytokines (IL-6 and IL-1β) and inflammatory mediators (NF-κB and iNOS) were assessed in intestinal tissues. The quantitative data in Fig 6 demonstrate significant inter-group variations in the levels of IL-6 (F(2, 9) = 20.930, *p* < 0.001) and NF-Κb (F(2, 9) = 7.151, *p* = 0.014). Post-hoc analysis showed that while CUMS+SE significantly drove up these inflammatory markers (*p* < 0.001 for IL-6; *p* = 0.021 for NF-κB), EE intervention effectively suppressed this pro-inflammatory response, bringing their expression back to control-like levels (*p* = 0.013 and *p* = 0.027, respectively; Figs 6D and E). Consistent with the protein data, RT-PCR analysis revealed significant group differences in *IL1B*, *NOS2*, and *NF-κB* mRNA expression (Figs 6H-J; F(2, 9) = 23.860, 6.893, and 9.371, respectively; all *p* < 0.05). The CUMS+SE group exhibited robust increases in all three markers relative to the CON group (*IL1B*: *p* = 0.003; *NOS2*: *p* = 0.019; *NF-κB*: *p* = 0.006), while EE intervention significantly attenuated these CUMS-induced elevations and shifted expression patterns toward control levels (*IL1B*: *p* < 0.001; *NOS2*: *p* = 0.037; *NF-κB*: *p* = 0.039 vs. CUMS+SE). Together, these findings demonstrate that CUMS induces intestinal inflammation, likely reflecting gut microbiota dysregulation. Significantly, EE effectively ameliorates these inflammatory alterations, supporting its protective role in modulating inflammation within the microbiota–gut–brain axis.

## Discussion

This study provides an integrated assessment of the effects of EE on depressive-like behaviors, gut microbial composition, and neurobiological alterations in a CUMS mouse model. Our findings show that chronic stress induces coordinated changes across behavioral, microbial, neurobiological, and intestinal domains. EE markedly attenuates stress‑induced alterations by reducing depressive-like behaviors, restoring gut microbial compositions, and improving intestinal barrier integrity and inflammation. Importantly, these therapeutic effects are likely driven by the synergistic—rather than strictly independent—contributions of the enriched physical environment, lower spatial density, and enhanced social interaction inherent to the EE paradigm.

Behavioral assessments demonstrated that the CUMS paradigm reliably induced depressive-like phenotypes, as evidenced by increased immobility in both the TST and FST [33,34]. These behavioral impairments are consistent with the well-established effects of chronic stress on emotional regulation and stress-coping behavior [35,36]. EE substantially ameliorated the observed behavioral deficits [37–40]. Animals housed in EE generally displayed greater adaptability, consistent with prior reports of enhanced environmental accommodation under enriched conditions [41–43]. Reflecting this improved adaptability, EE exposure was associated with reduced immobility in the TST. Although the immobility time in the FST did not decrease, these behaviors may be influenced by the greater environmental acclimation typically observed in EE animals [44–48]. Importantly, unaltered locomotor activity in the open field test (data not shown) excludes generalized motor deficits as a confounding factor. Taken together, these results indicate that EE contributed to reductions in immobility, supporting its overall impact on behavioral improvement.

At the neurobiological level, EE was associated with changes in molecular markers such as TPH and GR, which are key indicators of the stress response. The CUMS-related reduction in TPH expression in the dorsal raphe is consistent with previous findings of stress-induced disruptions in serotonergic regulation [21,49]. EE maintained TPH levels comparable to those of controls, consistent with reports that enriched environments can alleviate the abnormal serotonergic change [50]. Similarly, the increase in hippocampal GR expression following CUMS is known to reflect the hyperactivation of HPA axis signaling [51,52], and EE attenuated this effect, consistent with studies showing reduced glucocorticoid responsiveness under enriched conditions [53,54]. Collectively, these findings indicate that EE mitigated stress‑related molecular alterations in pathways commonly implicated in chronic stress models.

Gut microbiota findings further support the notion that EE can modulate gut microbiota composition [27]. CUMS mice exhibited significantly elevated alpha diversity (richness and evenness). While generally considered beneficial, such increases in stress models often reflect stress-responsive dysbiosis rather than ecological stability [55], consistent with clinical observations in depression [56]. EE prevented this increase, maintaining alpha diversity at levels similar to controls. These results indicate that EE helps preserve ecological stability in the gut microbiota under chronic stress. Beta diversity analysis also revealed distinct structural differences in microbial communities under CUMS, whereas EE maintained a community profile closer to that of the control group. Heatmap patterns supported this observation, suggesting that EE attenuated CUMS‑induced alterations in the microbial community.

At the phylum level, EE was associated with a higher relative abundance of *Bacteroidota* and lower abundance of *Firmicutes*, *Desulfobacterota*, and *Patescibacteria* compared with SE. *Bacteroidota* are commonly linked to carbohydrate fermentation and short‑chain fatty acid production, while increases in *Firmicutes* and *Desulfobacterota* have been reported in pro‑inflammatory or metabolically stressed states [57–60]. Changes in *Patescibacteria* have likewise been associated with microbial instability [61]. These patterns suggest that EE supported a more balanced microbial composition than that observed under standard housing conditions following CUMS [62,63]. At the genus level, EE partially reversed CUMS‑related alterations in key taxa. *Akkermansia*, associated with mucus‑layer maintenance and barrier support, was reduced by CUMS but returned to control‑like levels with EE [63]. Conversely, *Ruminococcus* increased under CUMS and declined with EE, consistent with reports linking particular *Ruminococcus* species to mucin degradation and pro‑inflammatory tendencies [64,65]. Overall, these findings indicate that EE mitigated stress‑related disruptions in gut microbial composition, supporting a more stable and functionally beneficial microbial community during chronic stress exposure [27,65,66].

CUMS altered the gut microbiota by decreasing epithelial‑supporting taxa and increasing taxa associated with mucosal stress. Similar patterns have been reported in other chronic stress models and are commonly linked to reduced barrier stability [67–70]. In line with this, the decrease in claudin‑1 expression observed here suggests impaired tight junction structure, consistent with evidence that stress‑induced dysbiosis compromises epithelial integrity [71–73]. EE prevented this reduction, maintaining claudin‑1 levels comparable to controls and supporting previous findings that enriched environments promote microbial communities favorable to barrier preservation [27,28,66]. Unlike claudin‑1, occludin expression increased following CUMS exposure. This differential regulation likely reflects their distinct roles: Claudin-1 is essential for barrier formation, whereas occludin primarily mediates junctional stabilization and regulatory signaling [74,75]. While chronic stress generally reduces tight junction proteins, the observed upregulation of occludin aligns with compensatory mechanisms reported during mucosal injury [76,77], suggesting an adaptive response to stabilize compromised junctions. Ultimately, EE restored claudin‑1 and maintained stable occludin levels, indicating preservation of both structural and regulatory components of the tight junction. To further validate these molecular findings, future in vivo functional permeability assays (e.g., FITC-dextran transit) will be valuable for confirming the macroscopic integrity of the gut barrier.

The inflammatory profile observed here also reflects patterns described in previous research. Elevated IL‑6, IL‑1β, NF‑κB, and iNOS expression under CUMS corresponds to mucosal immune activation, often observed when microbial imbalance and epithelial disruption co‑occur [72,78,79]. EE markedly reduced these signals, consistent with reports that EE can limit stress-related immune activation and promote gastrointestinal homeostasis [27,80]. Because intestinal inflammation is known to modulate central neurotransmission and HPA axis activity, the combined normalization of microbial composition, barrier-related proteins, and inflammatory markers under EE may contribute to the broader physiological effects observed in this model.

Overall, EE was associated with improvements in biological processes affected by chronic stress, including gut microbial composition, intestinal barrier status, inflammatory activity, and stress-related signals in the brain. EE consistently showed patterns indicative of reduced physiological disturbance under chronic stress. Although the parallel changes observed in the gut and the brain point to interactions within the microbiota–gut–brain axis, the precise causal pathways remain to be fully elucidated. Furthermore, this study did not assess functional microbial readouts, such as short-chain fatty acids. Future investigations incorporating metabolomics, fecal microbiota transplantation, and concurrent assessment of gut-derived and central markers will be necessary to mechanistically clarify the contribution of microbiota-related changes to the effects observed under enriched conditions [81,82]. A more detailed understanding of these relationships will advance the interpretation of EE-related processes in chronic stress models.

Despite these findings, several limitations of this study should be noted. First, the use of only male mice may limit the generalizability of our findings across sexes. Second, considering the high inherent variance in microbiome data, future studies with larger cohorts are warranted to further validate these microbial findings. Lastly, although the direct translation of rodent models to humans has inherent limitations, the core principles of EE hold substantial translational value. Indeed, adapted EE paradigms have already been successfully implemented in clinical settings, such as stroke rehabilitation, showing that enriched environments aid neurobehavioral recovery [83–85]. Therefore, when tailored to human contexts—by integrating structured exercise, cognitive stimulation, and social support—EE components could serve as a promising intervention for depression.

## Conclusion

EE reduced depressive‑like behaviors induced by CUMS and was accompanied by normalization of TPH and GR expression, indicating improved serotonergic and HPA‑axis regulation. EE also countered CUMS‑related changes in gut microbiota, producing a microbial profile closer to that of control animals. These microbiota differences were associated with preserved intestinal barrier function, reflected by higher claudin‑1 expression, and with reduced intestinal inflammatory markers, including IL‑6, IL‑1β, NF‑κB, and iNOS. These results highlight the promising potential of environmental enrichment as a non‑pharmacological intervention to restore multiple systems affected by chronic stress, inspiring further research and clinical application in the microbiota–gut–brain axis.

## Supporting information

S1 Fig Original uncropped membrane images for Western blot analysis. The original raw images of the membranes used for (A) Claudin-1, (B) Occludin, (C) NF-κB, (D) IL-6, and (E) β-actin are presented. Red dashed boxes indicate the representative bands used in the main figures. Molecular weight markers (Ladder) are indicated on the left of each membrane. (A) For Claudin-1, the non-specific band at ~25 kDa is identified as the IgG light chain. (B-E) For multiple detections from a single electrophoresis run, membranes were horizontally cut based on molecular weight markers prior to antibody incubation.
(DOCX)

S2 Fig Original uncropped agarose gel images for RT-PCR analysis. The original raw images of the agarose gels for (A) *CLDN1*, (B) *IL1B,* (C) *NOS2*, (D) *NF-κB*, and (E) *ACTB* are presented. DNA ladders are indicated on the left of each gel to confirm the target product sizes. Red dashed boxes indicate the representative bands used in the main figures. (E) *ACTB* was used as an internal loading control to ensure equal template concentration across all samples.
(DOCX)

## Abbreviations

MDD: Major depressive disorder
HPA: Hypothalamic-Pituitary-Adrenal
IL-6: Interleukin-6
IL-1β: Interleukin-1β
iNOS: Inducible nitric oxide synthase
NF-κB: Nuclear factor-κB
TNF-α: Tumor necrosis factor-α
TPH: Tryptophan hydroxylase
GR: Glucocorticoid receptor
CUMS: Chronic unpredictable mild stress
EE: Environmental enrichment
TST: Tail suspension test
FST: Forced swim test
