# Supplementary material for: Environmental enrichment attenuates chronic stress–induced disruptions in the gut microbiota, intestinal barrier, and brain
Source: PLoS One. 2026 Aug 14;21(8):e0355857. doi: 10.1371/journal.pone.0355857 (PMC13475964; doi:10.1371/journal.pone.0355857)
Supplement: Supplementary file 1 [file pone.0355857.s001.docx]

**S1 Fig. Original uncropped membrane images for Western blot analysis.** The original raw images of the membranes used for (A) Claudin-1, (B) Occludin, (C) NF-κB, (D) IL-6, and (E) β-actin are presented. Red dashed boxes indicate the representative bands used in the main figures. Molecular weight markers (Ladder) are indicated on the left of each membrane. (A) For Claudin-1, the non-specific band at ~25 kDa is identified as the IgG light chain. (B-E) For multiple detections from a single electrophoresis run, membranes were horizontally cut based on molecular weight markers prior to antibody incubation.
