# Supplementary material for: Environmental enrichment attenuates chronic stress–induced disruptions in the gut microbiota, intestinal barrier, and brain
Source: PLoS One. 2026 Aug 14;21(8):e0355857. doi: 10.1371/journal.pone.0355857 (PMC13475964; doi:10.1371/journal.pone.0355857)
Supplement: Supplementary file 2 [file pone.0355857.s002.docx]

**S2 Fig. Original uncropped agarose gel images for RT-PCR analysis.** The original raw images of the agarose gels for (A) *CLDN1*, (B) *IL1B*, (C) *NOS2*, (D) *NF-κB*, and (E) *ACTB* are presented. DNA ladders are indicated on the left of each gel to confirm the target product sizes. Red dashed boxes indicate the representative bands used in the main figures. (E) *ACTB* was used as an internal loading control to ensure equal template concentration across all samples.
